# Samba virus: a novel mimivirus from a giant rain forest, the Brazilian Amazon

**DOI:** 10.1186/1743-422X-11-95

**Published:** 2014-05-14

**Authors:** Rafael K Campos, Paulo V Boratto, Felipe L Assis, Eric RGR Aguiar, Lorena CF Silva, Jonas D Albarnaz, Fabio P Dornas, Giliane S Trindade, Paulo P Ferreira, João T Marques, Catherine Robert, Didier Raoult, Erna G Kroon, Bernard La Scola, Jônatas S Abrahão

**Affiliations:** 1 Departamento de Microbiologia, Universidade Federal de Minas Gerais, Laboratório de Vírus, Av. Antônio Carlos, 6627 Pampulha, Belo Horizonte, MG Zip Code 31270-901, Brazil; 2 Departamento de Bioquímica e Imunologia, Instituto de Ciências Biológicas, Universidade Federal de Minas Gerais, Av. Antônio Carlos, 6627 Pampulha, Belo Horizonte, MG CEP 31270-901, Brazil; 3 Unité de Recherche sur les Maladies Infectieuses et Tropicales Emergentes (URMITE), UM63 CNRS 7278 IRD 198 INSERM U1095, Faculté de Médecine, Aix-Marseille Université, Marseille, France

**Keywords:** Mimiviridae, DNA virus, Giant virus, NCLDV, Virophage, Amazon, Brazil

## Abstract

**Background:**

The identification of novel giant viruses from the nucleocytoplasmic large DNA viruses group and their virophages has increased in the last decade and has helped to shed light on viral evolution. This study describe the discovery, isolation and characterization of Samba virus (SMBV), a novel giant virus belonging to the *Mimivirus* genus, which was isolated from the Negro River in the Brazilian Amazon. We also report the isolation of an SMBV-associated virophage named Rio Negro (RNV), which is the first *Mimivirus* virophage to be isolated in the Americas.

**Methods/results:**

Based on a phylogenetic analysis, SMBV belongs to group A of the putative *Megavirales* order, possibly a new virus related to *Acanthamoeba polyphaga mimivirus* (APMV). SMBV is the largest virus isolated in Brazil, with an average particle diameter about 574 nm. The SMBV genome contains 938 ORFs, of which nine are ORFans. The 1,213.6 kb SMBV genome is one of the largest genome of any group A *Mimivirus* described to date. Electron microscopy showed RNV particle accumulation near SMBV and APMV factories resulting in the production of defective SMBV and APMV particles and decreasing the infectivity of these two viruses by several logs.

**Conclusion:**

This discovery expands our knowledge of *Mimiviridae* evolution and ecology.

## Background

The discovery of the *Acanthamoeba polyphaga mimivirus* (APMV), arguably the most elusive member of the nucleocytoplasmic large DNA virus (NCLDV) group and the first discovered member of the *Mimiviridae* family, revived discussions regarding the evolution and origin of the viruses, as well as the differentiation between viruses and living organisms [1]. The complexity of NCLDVs in terms of genome size, particle size and metabolic capabilities (such as their role in photosynthesis and apoptosis) has challenged many concepts in virology [2]. However, it was the discovery of APMV that spotlighted NCLDVs [3]. This novel member of the NCLDV group, which belongs to the proposed *Megavirales* order, is extremely large and complex and contains genes related to translational activity, which were hitherto considered to be exclusive to cellular organisms [4].

The family *Mimiviridae* is comprised of double stranded DNA viruses up to 750 nm of diameter with genomes containing up to 1.2 Mb. The mimiviruses are some of the most complex viruses known to date and are important members of the NCLDV group [5]. One of this family’s key members is the *Mimivirus* genus, whose type species is APMV. APMV was isolated in 1992 from a water cooling tower at a hospital in Bradford, England and was investigated as a putative etiological agent of pneumonia [6]. The APMV particle is composed of a core, internal membrane, a capsid and external fibrils. The capsid has semi-icosahedral pentagonal symmetry and with a star-shaped structure called the star gate [7,8].

To date, members of the *Mimiviridae* family have been isolated in England, France, Tunisia, Chile and a few other countries [3,4,9]. Additionally, DNA from these viruses was identified in the Sargasso Sea and other ocean samples using metagenomic approaches [10-12]. While APMV was isolated from a cooling tower at a hospital in England, *Megavirus chilensis* (MCHV), a putative new species of the *Mimiviridae* family, was isolated on the coast of Chile, indicating that members of this family can be found in a range of environmental conditions [3,4].

*Acanthamoeba castellanii mamavirus* (ACMV), a strain of APMV, was isolated from a water cooling tower at a hospital in France, together with another virus, *Sputnik virus* (SNV) [13]. SNV decreased the infectivity of ACMV in cultured *Acanthamoeba castellanii*, leading to its classification as the first virophage [13]. Since then, other viruses with similar biological activity to SNV have been isolated from other NCLDVs, consolidating the emerging concept of virophages [14,15].

*Mimiviridae* family viruses have been isolated in different countries from aquatic environments that display as wide range of temperatures and salinity [3,4,8,9]. However, to date, only one *Mimivirus* has been isolated in the Americas [4]. In this work we report the isolation, biological characterization, genome sequencing and annotation of the giant Samba virus (SMBV) and its associated virophage Rio Negro virophage (RNV), both isolated from the Brazilian Amazon, known to be one of the most biodiverse ecosystems in the world.

## Results and Discussion

### Collection area data

**Figure 1:**
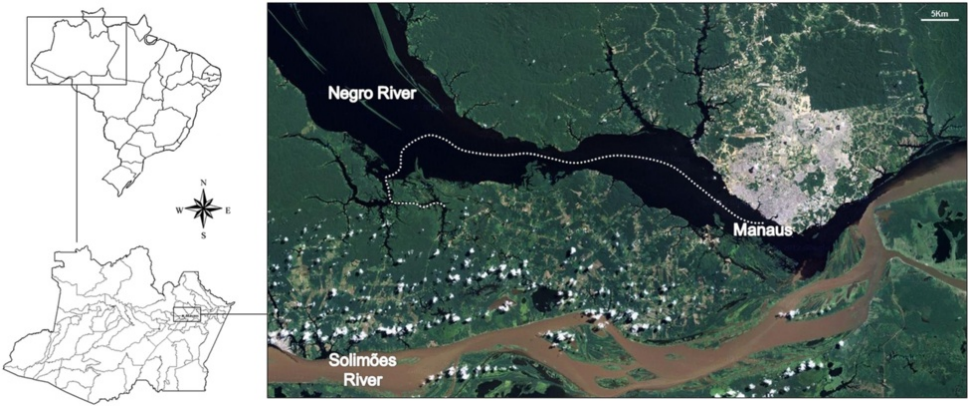
**Location of the collections.** Water samples were collected from the Negro River, which is located in the Brazilian Amazon.

We decided to explore the Brazilian Amazon with the goal of isolating giant viruses. Although the biodiversity of the Amazon is well known, there are no studies regarding the potential presence of giant viruses in this environment. We collected surface water samples in October of 2011 from the Negro River (Figure 1), an affluent of the Amazon River, which is mostly in Brazilian territory. This river is acidic due to large amounts of dissolved organic substances. Rainwater flow carries organic acids from decomposing vegetation residue to the river, resulting in its dark color (“Dark River” means “Rio Negro” in Portuguese). A total of 35 water samples were collected along a 65 km route beginning at Manaus (3°6’S 60°1’W), the capital city of Amazonas State, and stored at 4°C. The samples were collected from surface water, near aquatic plants, near indigenous tribal areas, and from small Negro River affluents.

### SMBV: isolation of a giant virus from the Negro River in the Amazon, Brazil

**Figure 2:**
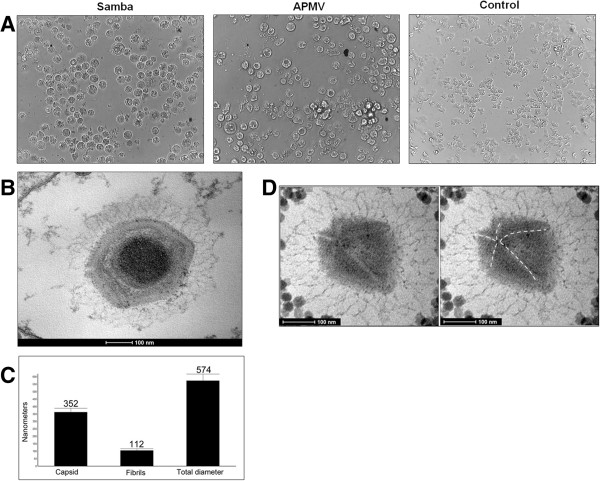
**Isolation and characterization of SMBV. A)** SMBV-induced CPEs in an ***A***. *castellanii* monolayer, which are similar to APMV-induced CPEs; **B)** SMBV particle visualized by transmission electronic microscopy; **C)** Morphometry of the SMBV particle, which has an average diameter of approximately 574 nm (a total of 50 particles images were analyzed); **D)** Detail of the star-gate structure (visualized by electronic microscopy), which is also present in APMV.

Samples collected from the Negro River were enriched in rice water medium and filtered. Isolation was carried out by growth in *A. castellanii* monolayers. After two blind passages, a sample collected near Manaus (3°7′34.00” S 60°4′25.00” W) induced cytopathic effects (CPE), including cell rounding and lysis after 2 days (Figure 2A). Samples collected in parallel were assayed by real-time PCR [16] and were positive for the amplification of the mimivirus helicase gene DNA, suggesting the presence of a giant virus. This Amazonian virus was named Samba virus (SMBV).

To characterize this potential new giant virus, SMBV was grown and purified in *A. castellanii* as described previously [3], and *A. castellanii* cells infected with SMBV at a TCID_50_ per cell rate of 10 were analyzed by electron microscopy (EM). Uninfected amoebae were used as controls. After seven hours of infection, EM images revealed the presence of giant viruses with multi-layered capsids covered with fibrils (Figure 2B). The capsids averaged 352 nm in diameter, the fibrils averaged 112 nm in length, and the average diameter of the particles was 574 nm (Figure 2C), making SMBV the largest virus ever isolated in Brazil (a total of 50 particles images were analyzed) and the first mimivirus isolated in this country. Actually, the size of the particles is likely to be significantly larger since chemical preparation might be related to particles shrinkage. In some of the images we detected a hypothetical star-gate structure, which has been described in other giant viruses (Figure 2D). We next observed purified virus using a light microscope. Remarkably, we were able to detect several particles at 1000× magnification on agarose surface, similarly to APMV.

**Figure 3:**
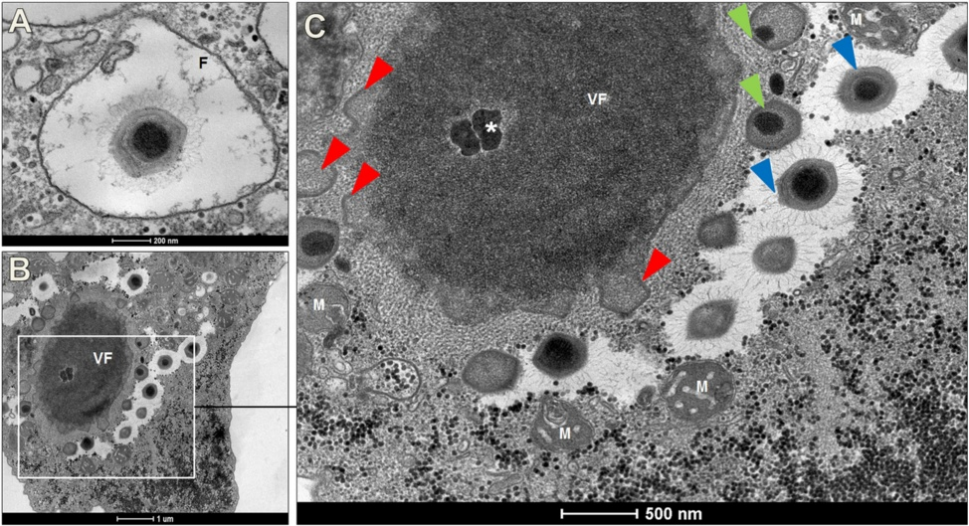
**SMBV replication cycle.** Replication of SMBV in *A. castellanii* observed by transmission electronic microcopy. **A)** SMBV enters amoebae by phagocytosis and remains within the phagosome; **B)** Giant viral factories are present within the amoebal cytoplasm; **C)** Morphology of SMBV near the viral factory: early morphogenesis (red arrows), intermediate morphogenesis (green arrows) and late morphogenesis (blue arrows). * = Viral seed; M = mitochondria, VF= Viral factory.

EM images obtained at different infection times suggest that SMBV entry is mediated by phagocytosis (Figure 3A). TEM images of SMBV in amoebae revealed a large viral factory occupying a large portion of the amoeba cytoplasm (Figure 3B). We observed viral morphogenesis in association with the viral factories, which presented particles in the early (Figure 3C, red arrows), intermediate (Figure 3C, green arrows) and final stages (Figure 3C, blue arrows) of assembly. Therefore, SMBV life cycle is very similar to that described to APMV.

### Isolation and characterization of RNV, the virophage associated with SMBV

**Figure 4:**
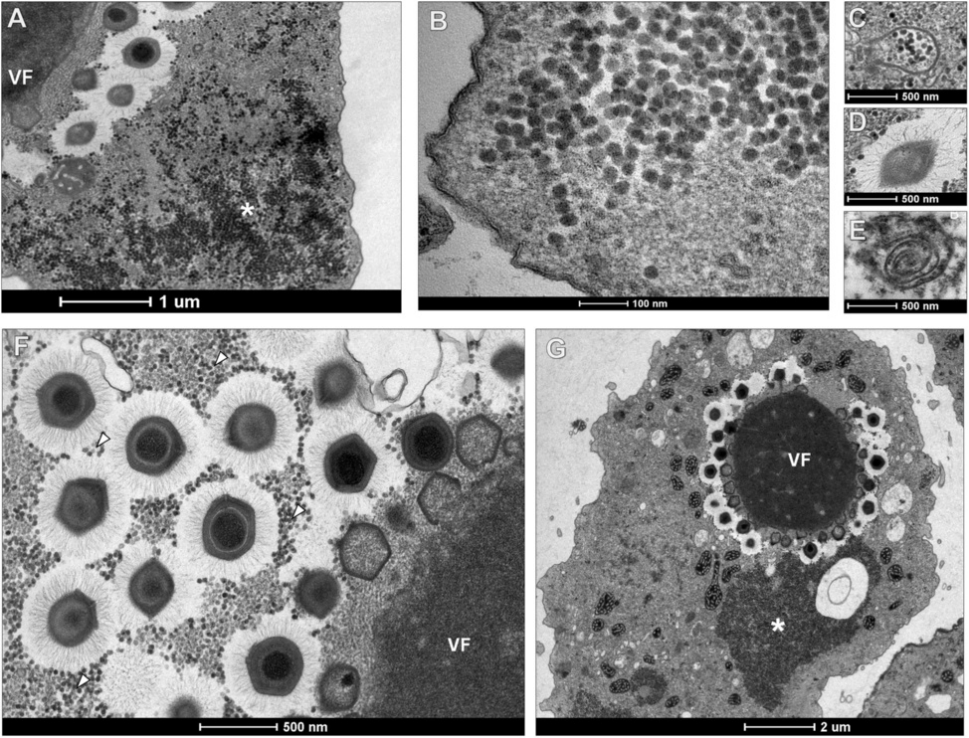
**EM of the virophage RNV.** Electron microscopy of virophage accumulation in the cytoplasm of *A. castellanii* infected with SMBV and APMV. **A**, **B)** Significant accumulation of virophage particles (indicated by an asterisk) around the viral factories and SMBV particles; **C**, **D**, **E)** SMBV particles with defective morphology as a result of infection by virophage; **F**, **G)** Virophage isolated from SMBV are also able to associate with APMV and accumulate near the viral factories, leading to the formation of defective viral progeny. VF = viral factory.

While analyzing the EM images, we were intrigued by the presence of a myriad of ~35 nm hexagonal-shaped particles in amoebas infected with SMBV (Figure 4A and 4B). These particles were adjacent to the SMBV viral factories, and some of them associated with the viral particles during the final assembly phase. A careful examination of infected amoebas revealed the presence of atypical SMBV particles, including defective capsids wrapped around small (roughly 35 nm) particles (Figure 4C), lemon-shaped particles (Figure 4D), and defective spiral capsids (Figure 4E). Because previous studies have described similar phenomena in amoebae co-infected with ACMV and SNV, we decided to investigate the nature of these small particles. Real-time PCR for the SNV capsid gene was performed using SMBV-infected amoeba as template, and the expected fragment was amplified from those cells, but not from the controls (water or uninfected amoebas). A primer pair was designed to amplify a large fragment of the capsid gene based on the consensus sequence of virophage capsid genes available in GenBank. An amplicon was generated from SMBV-infected amoebas and then confirmed by sequencing.

To analyze the influence of the virophage on giant virus replication, purified SMBV were diluted and filtered. The aliquoted virophage solution was stored at -80°C until use. An *A. castellanii* monolayer was co-infected with APMV (TCID_50_ per cell rate of = 10) and 100 μl of the solution containing the undiluted virophage isolated from SMBV. After 16 hours, we analyzed the cells by EM. Remarkably, virophage particles derived from SMBV were observed in association with APMV particles during viral assembly (Figure 4F). We also observed defective APMV particles similar to those seen in SMBV and virophage co-infected amoebas. Large areas of virophage accumulation covering more area than the APMV factories themselves were observed in some of the co-infected amoebas (Figure 4G).

**Figure 5:**
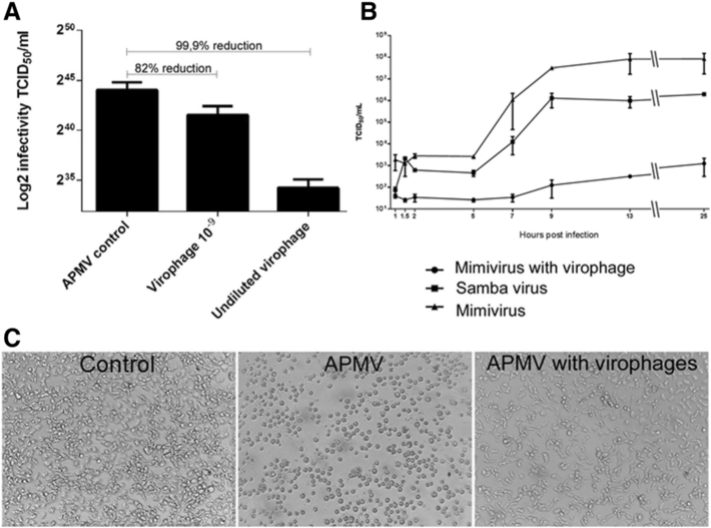
**Reduction of viral infectivity by co-infection with virophage.** Evaluation of the biological activity of virophage isolated from SMBV through viral infectivity reduction assays: **A)** Titration of APMV in *A. castellanii* after co-infection with RNV showed a reduction in viral titer by more than 80% compared to the control; **B)** A one-step growth curve of APMV in the presence of virophage showed that RNV drastically reduces the ability of APMV to multiply in *A. castellanii*, leading to a significant decrease in viral titer compared to control curves generated with SMBV naturally associated to RNV and APMV in the absence of virophage. **C)** Reduction in APMV-induced CPEs in amoeba when co-infected with virophage, visualized by light microscopy (after 12 hours).

To quantify the SMBV virophage inhibition of giant virus replication, *A. castellanii* was infected with APMV at a TCID_50_ per cell rate of 1 and superinfected with 100 μl of virophage solution, undiluted or diluted to 10^-9^. The TCID_50_ was determined after 48 hours by observing the APMV-induced CPEs in *A. castellanii* (Figure 5A). In parallel, we performed one-step growth curves (0 to 25 hours) of APMV, SMBV (naturally associated to RNV) and APMV + RNV (Figure 5B). In both assays, RNV caused a decrease in APMV titers, ranging from 2 log_10_ at early timepoints to 5 log_10_ at 25 hours post infection. The inhibitory effect of the virophage could also be observed by light microscopy. Instead of the rounding induced by APMV replication, co-infected cells exhibit milder CPEs at the same timepoint, resembling the control cells more than the APMV-infected cells (Figure 5C). No CPEs were observed in amoebas inoculated only with the virophage RNV.

### SMBV genome

**Figure 6:**
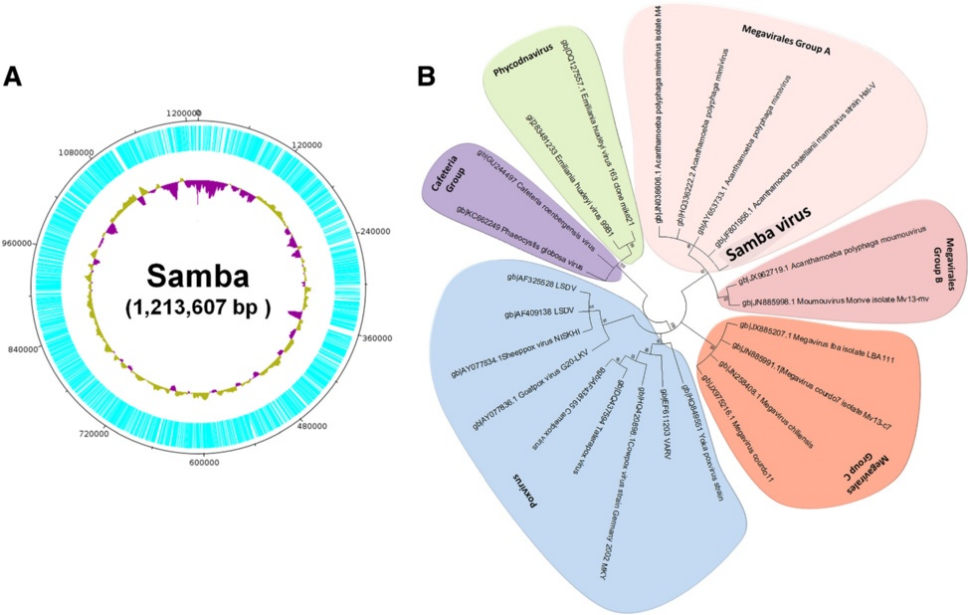
**Samba virus genome (circular) and phylogeny: (A) Estimated Samba virus genome size assembled using the Perl**-**based program ABACAS 1.3.1-1.** The predicted ORFs are highlighted in blue, revealing a very low proportion of noncoding DNA. The innermost circle indicates the G-C content. Regions with a G-C content higher than the average (28%) are highlighted in green, and lower than average in purple. **(B)** Phylogenetic tree (neighbor joining, 1,000 bootstrap replicates, n. differences model) based on the ribonucleotide reductase predicted amino acid from the Samba virus and other nucleocytoplasmic large DNA viruses. The Samba virus clusters with members of Megavirales group A, which is comprised of Mimivirus and Mamavirus. The tree is unrooted. The values near the branches are bootstrap values calculated using the MEGA 4 program and are used as confidence values for the tree branches.

**Figure 7:**
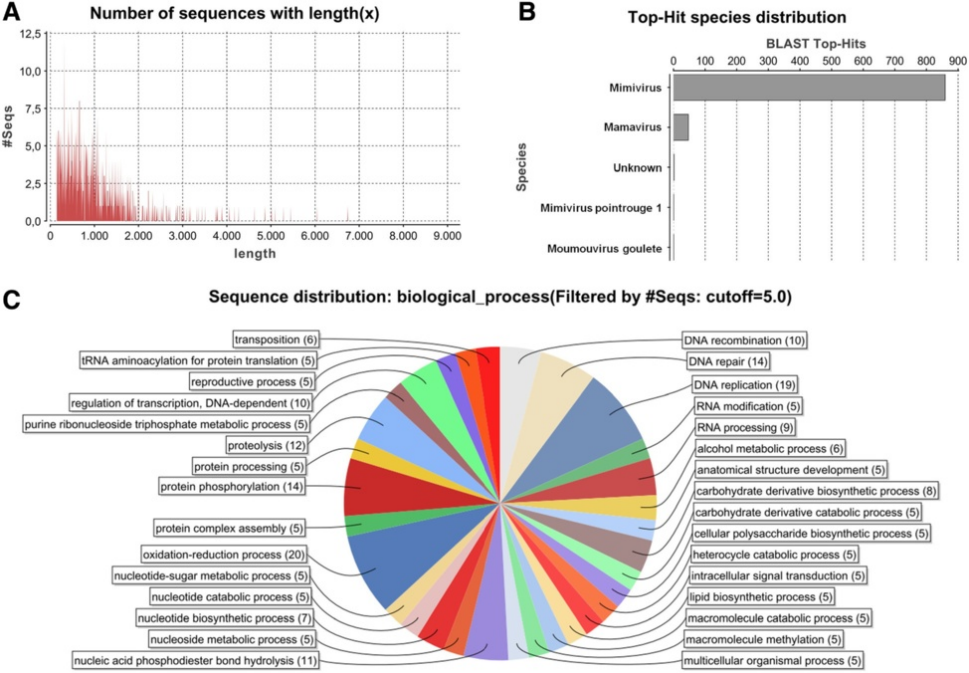
**Genomic data.** Samba virus genome characterization performed using the java-based free software Blast2go (available at http://www.blast2go.com/b2ghome): **(A)** Graphical distribution of the length of the predicted Samba virus genes, showing the predominance of small ORFs ranging mainly from 150 to 1500 nucleotides. **(B)** Graphical distribution of the sequence similarity search for the predicted Samba virus genes. The greatest number of hits were found in Mimivirus, followed by Mamavirus. **(C)** Biological processes assigned to the predicted Samba virus genes, showing a large proportion of genes involved in nucleic acid processing and cellular metabolism, as well as viral morphogenesis and intracellular regulation.

After sequencing, assembly and annotation, we obtained a partial SMBV genome (scaffold) of 1,213,607 bp (Figure 6A), which is comparable in size to the largest genomes described for mimiviruses to date (Figure 6B). We achieved approximately 98.8% coverage of the genome (considering APMV covered orthologous) (Genbank access number: KF959826). The SMBV genome has a GC content of 27.0% (Figure 6A) and is approximately 50,000 bp larger than the APMV genome. A total of 938 ORFs, ranging in size from 150 to 8835 bp (Figure 7A), were annotated as putative genes. The average ORF size is 1001.8 bp. Using these ORFs, a gene similarity search was conducted using the Blast2GO platform. Many of the ORFs within the SMBV genome had only been found previously in viruses from the *Mimiviridae* family, including those which putatively encode proteins with roles in protein translation or DNA repair. Overall, SMBV shared the most ORFs in common APMV (~91%), followed by ACMV (~6%), *Mimivirus pointrouge* (~0.5%), *Moumouvirus goulette* (~0.5%) and others (~2%) (Figure 7B). An analysis of the SMBV genome identified 19 ORFs related to DNA replication, 10 involved in DNA recombination, 14 linked to DNA repair and five related to tRNA aminoacylation, which is important for protein translation (Figure 7C). We also identified 264 domains that are putatively related to protein binding and 200 domains linked to catalytic activity (Additional file 1: Figure S1A). The sequence similarity between SMBV ORFs and the homologous genes in GenBank ranged from 35 to 100%, with many candidates showing 50 to 60% similarity, indicating that SMBV genome has many unique features (Additional file 1: Figure S1B). Although 47.1% of the predicted ORFs in the SMBV genome have homologs in other giant virus genomes, they are not homologous to other organisms and are therefore considered hypothetical proteins (Additional file 1: Figure S1C). Interestingly, a dotplot analysis of SMBV vs. APMV ORFs (gene synteny) revealed that, although the majority of SMBV genes are present in the same genome locus described for APMV, many ORFs are inverted or in distinct loci, especially those in the terminal regions (Additional file 2: Figure S2).

### SMBV phylogeny

To determine which viruses SMBV is most closely related to, we performed the following analyses: [1] we constructed phylogenetic trees by aligning the ribonucleotide reductase (Figure 6B) and helicase gene (data not shown) sequences from SMBV with GenBank sequences and [2] we constructed a Venn diagram showing a presence-absence analysis of ORFs from SMBV and related viruses (Additional file 3: Figure S3). Both phylogenetic trees show that SMBV clusters with members of *Megavirales* order group A (MGA), which includes APMV (the prototype of the family) and ACMV (Figure 6B). MGA was most closely related to *Megavirales* group B (MGB), which includes *Acanthamoeba polyphaga moumouvirus* (APMOUV), and was distinct from *Megavirales* group C, which includes MCHV and other viruses. Other NCLDV family members were also included in the analysis, and their position in the phylogenetic tree generated in this study corroborates previous phylogenetic studies. The phylogenetic trees were constructed by MEGA 4.1 using neighbor joining, maximum parsimony and other methods with 1,000 bootstrap replicates (n. differences model) based on the ribonucleotide reductase predicted amino acid from the SMBV and other nucleocytoplasmic large DNA viruses. The same tree topology was observed for all phylogenetic approaches.

Next, a Venn diagram (Additional file 3: Figure S3) was constructed using the predicted ORFs from SMBV and the ORF data set from its closest relatives, including APMV and ACMV (from the MGA group), APMOUV (from the MGB group) and MCHV (from the MGC group). The SMBV ORFs were aligned with the ORF data set of each viral counterpart using the Blastn all-against-all method feature of Blastall 2.2.9. Only hits with an e-value ≤ 10-5 were considered valid. The diagram (Additional file 3: Figure S3) shows that nine SMBV ORFs did not appear in other viral genomes. Of these, ORF-L331 appears to contain a signal-peptide cleavage site, and ORF-R518 exhibits a transmembrane domain. The remaining ORFans had no known function or domain. SMBV shares 925 ORFs with APMV, 909 with ACMV, 346 with MCHV and 312 with APMOUV. SMBV, APMV and ACMV (MGA strains) share 503 ORFs. SMBV, MGA and APMOUV (MGB) share 61 ORFs, while SMBV, MGA and MCHV (MGC) shared 93 ORFs. All of the viruses analyzed share a core repertoire of 249 ORFs (Additional file 3: Figure S3).

### RNV virophage sequence analysis

The RNV capsid gene was also sequenced and analyzed (primers: 5’ATGTCTAATTCAGCTATTCCTCTTA3’ and 5’TCACATTTTAAGTTCTTTTCTCAAT3”). We then manually aligned the sequence with conserved virophage sequences from GenBank and used Modeltest software to determine which model of evolution was most appropriate for our analysis. Phylogenetic trees based on the capsid gene sequence were constructed using MEGA 4.1 with neighbor joining, maximum parsimony and other methods with 1,000 bootstrap replicates. The RNV capsid sequence was deposited in GenBank (KJ183141). Our results show that the RNV capsid gene shares 100% identity with SNV and high identity with other SNV genes. Therefore, RNV clusters with SNV-like viruses and does not cluster with Mavirus or the Organic Lake Virophage (Additional file 4: Figure S4). Unfortunately, it was not possible to recover RNV contigs from SMBV sequencing data.

## Conclusions

Giant viruses exhibit strikingly large genome and particle sizes, and have shed light on the evolution of large DNA viruses [3,4,17-25]. This study describes the discovery, isolation and characterization of Samba virus, a novel mimivirus, from the Negro River in the Brazilian Amazon. SMBV is phylogenetically related to *Megavirales* order group A, which comprises APMV and ACMV, and contains one of the the largest genome described for this group.

However, despite its close relationship to APMV, the SMBV genome is 50,000 bp longer than APMV and is unique for its ORF content. The SMBV and APMV genome alignment showed a high degree of synteny among the conserved genes, although we did observe some inversions (Additional file 2: Figure S2). Many of the SMBV ORFans exhibited homology to APMV sequences, although they did not appear to correspond to ORFs in the APMV genome. This may be explained by the use of different bioinformatics tools for ORF prediction during the genome annotation. The difference of 50,000 bp between the SMBV and APMV genomes is most likely due to intergenic regions and/or ORF size variation. Regarding the SBMV cycle of life, our data showed viral entry by phagocytosis and morphogenesis in association with the viral factories. All steps of viral morphogenesis resembled those described previously to APMV and other mimiviruses [3,4].

The presence of a *Mimiviridae* family virus and virophage in the Negro River correlates well with previous studies that indicate the presence of these viruses in aquatic ecosystems [10,11,24,25]. SMBV appeared to tolerate the virophage better than APMV, although we have not yet been able to generate virophage-free cultures. Nevertheless, these results suggest SMBV has developed mechanisms to circumvent the virophage. However, future studies are necessary to confirm this hypothesis. Interestingly, recent data suggested that virophages may be important for control of giant viruses and amoeba populations [26,27].

The discovery and characterization of SMBV and its virophage raise new questions regarding the role of these viral agents in microbial ecology. The Amazon rain forest contains a striking diversity of flora and fauna, although little is known regarding the virosphere in this environment. The discovery of SMBV in the Amazon corroborates that these fascinating viruses are ubiquitous and that their isolation and characterization can yield important insights into their life cycle and complexity.

## Methods

### Cell line

*Acanthamoeba castellanii* (ATCC 30010) was kindly provided by the Laboratório de Amebíases (Departamento de Parasitologia, ICB/UFMG), and cultivated in PYG medium (Visvesvara & Balamuth, 1975) supplemented with 7% fetal bovine serum (FBS) (Cultilab, Brazil), 200 U/mL penicillin (Cristália, Brazil), 50 μg/mL gentamycin (Sigma, USA) and 2.5 μg/mL amphotericin B (Sigma, USA) at 28°C.

### Virus

APMV was obtained from a cooling tower at a hospital in Bradford (England) in 1993 and characterized in 2003 [3]. APMV was used as a control for the molecular and biological assays. For viral replication, *A. castellanii* cultures were inoculated at a TCID_50_ per cell rate of 0.1 and incubated in sealed bottles at 32°C.

### Sample collection and virus isolation

The water samples were collected from Negro River, Amazonas and stored at 4°C overnight. The sample collections were performed with permission of Instituto Chico Mendes (ICM) – protocol numbers: 34293-1 and 33326-2. The field studies did not involve endangered or protected species. Then, 500 μl of each sample was added to 4.5 mL of autoclaved rice and water medium made with 40 rice grains in 1 liter of water [4]. The samples were stored for 20 days in the dark at room temperature [4], then 5 × 10^3^*A. castellanii* trophozoites were added, and the samples were re-incubated under the same conditions for 10 days. After the enrichment process, samples were pooled in groups of five (totaling seven pools), and filtered through a 1.2 μm membrane to remove impurities and a 0.2 μm membrane to retain giant viruses. The samples were then subjected in parallel to real-time PCR and to viral isolation in *A. castellanii*.

### Viral titration

The virus titration was performed in 96-well plates containing approximately 4 × 10^4^*A. castellanii* per well in 200 μL of PYG medium supplemented with 7% FCS. The viral samples were serially diluted (from 10^-1^ to 10^-9^) in 50 μL of PBS, 150 μL of PYG medium with 10% FCS was added to each well, and the plates were incubated. CPEs such as rounding, loss of motility and trophozoite lysis were monitored daily in each well. After five days of incubation, the titer (TCID_50_) was calculated as described by Reed and Muench [28].

### Viral purification

To purify the giant viruses, cell lysates were centrifuged at 900 g for 5 minutes at 4°C. The supernatant was transferred to a fresh tube, and the pellet was subjected to three cycles of freezing and thawing to release virus trapped in the trophozoites. The lysate was homogenized in 10 mL of PBS and subjected to an additional two rounds of 50 homogenization cycles in a Dounce (Wheaton, USA). The supernatant and cell lysates were then filtered through a 2 μm filter (Millipore, USA). This filtrate was slowly dripped over 10 mL of a 22% sucrose solution (Merck, Germany) and ultra-centrifuged in a Sorvall Combi at 14,000 rpm for 30 minutes at 4°C. The pellet was homogenized in 500 μL of PBS. Aliquots of the virus were stored at -80°C and then titrated. To purify the virophages, 5 vials containing 50 μl each of previously purified infectious SMBV particles were diluted in 20 mL of PBS and filtered through 0.2 μm filters, which retain the giant viruses but not the virophages. The flow-through was collected and used in the biological assays.

### Growth curve

The infectivity assays were performed by inoculating *A. castellanii* with the viral samples at an m.o.i of 10. The viruses were allowed to adsorb for 1 hour, and then the cells were washed with PBS and incubated at 32°C. At 1, 2, 4, 6, 8, 12, 24 and 48 hours, the cultures were frozen and thawed three times and titrated.

### Inhibition of APMV growth after infection with isolated virophages

*A. castellanii* was infected with APMV at a TCID_50_ per cell rate of 1 and then superinfected with 100 μl of virophages, undiluted or diluted to 10^-9^. After 48 hours of infection, the infectivity (TCID_50_) was determined by observing APMV-induced CPEs and by titration (TCID_50_) in *A. castellanii* for five days.

### PCR

The PCR assays were performed using primers constructed based on the helicase gene from APMV gene (primers: 5’ACCTGATCCACATCCCATAACTAAA3’ and 5’GGCCTCATCAACAAATGGTTTCT3’). The PCR contained 2.0 mM MgCl_2_, 10 mM nucleotides (dATP, dCTP, dGTP, dTTP), 2 U of Taq DNA polymerase (Promega, USA), 2.0 μl 10× Taq polymerase buffer, 4 mM primers, and 2 μl of the sample in a 20 μl total reaction volume. The amplification was performed according to the conditions recommended for StepOne (Applied Biosystems, USA) with an annealing temperature of 60°C. The PCR-amplified DNA was fractionated on a 1% agarose gel at 100 V and stained with Gel Red (Biotium, USA). The real-time PCR was performed using a commercial mix (Applied Biosystems, USA), primers (4 mM each) and 1 μl of sample in each 10 μl reaction.

### Transmission electron microscopy

*A. castellanii* were infected at a TCID_50_ per cell rate of 10. Uninfected amoebae were used as controls. After 7 hours of infection, the amoebae were washed twice with 0.1 M phosphate buffer (pH 7.4) and fixed with 2.5% glutaraldehyde (grade I) in 0.1 M phosphate buffer (pH 7.4) (Electron Microscopy Sciences, Germany) for one hour at room temperature. The amoeba monolayer was scraped from the plates and recovered by centrifugation at 900 g for 5 minutes. The amoebae were postfixed with 2% osmium tetroxide and embedded in EPON resin. Ultrathin sections were stained with 2% uranyl acetate and examined using a Tecnai G2-Spirit FEI 2006 transmission electron microscope operating at 80 kV at the Microscopy Center, UFMG, Brazil.

### Sequencing analysis

The SMBV genome was sequenced using a 454 platform (Roche). For genome assembly, we used the read mapping approach implemented by the CLC Genomics Workbench 5.5.1 program to generate contig sequences, and the Perl-based genome assembly tool ABACAS.1.3.1 (algorithm-based automatic contiguation of assembled sequences) that yielded a partial genome (scaffold) of 1,213,607 bp (KF959826) (draft). The open-reading frames (ORF) were predicted using the Markov-based methods employed by Glimmer3 and FGENES. We also transferred annotations from a closely related genome using the Rapid Annotation Transfer Tool. The predictions were manually curated, and the ORFs were assigned a final identity. ORFs smaller than 150 bp were ruled out. Finally, 938 ORFs were annotated as putative genes. A gene similarity search was conducted using Blast2GO.

## Competing interests

The authors declare that they have no competing interests.

## Authors’ contributions

RKC, PVB, FLA, ERGRA, LCFS, JDA and FPD performed experiments (collection, isolation, biological and molecular characterization). GST, PCPF, JTM, CR, DR, EGK, BS and JSA designed and analyzed the results. All authors read and approved the final manuscript.

## Supplementary Material

Additional file 1 **Genomic data 2: Samba virus genome characterization performed using the java-based free software Blast2GO (available at**http://www.blast2go.com/b2ghome**). (A)** Graphical distribution of the functions and domains of predicted Samba virus genes. Most of the functions are related to catalytic and binding activities. **(B)** Graphical representation of the similarity of Samba virus genes to sequences available in the data bank of the Blast2GO program. The analysis showed a broad distribution of similarity ranging between 50-60%, with a peak near 100%. **(C)** Graphical depiction of Samba virus genes with or without functional annotation (IPS – InterProScan) and Samba genes grouped into orthologous groups (GO – Gene Ontology). Click here for file

Additional file 2 **Dotplot of SMBV vs. APMV ORFs – MUMMER 3.0 software.** Dots represent the predicted ORFs. The red dots = plus-plus ORFs, and the blue dots = inverted ORFs. Although most of the SMBV genes are present in the same genome locus described for APMV, many ORFs are inverted or located in distinct loci, especially those present in terminal regions. Click here for file

Additional file 3 **Venn diagram: Venn diagram of predicted Samba virus genes relative to other Mimiviridae genomes.** APMV – Acanthamoeba polyphaga mimivirus; Megavirus – Megavirus chilensis; Moumouvirus - Acanthamoeba polyphaga moumouvirus; Mamavirus – Acanthamoeba castellanii mamavirus. Boxes show each gene included in the intersections. “R” (right) refers to genes that are transcribed in the positive sense, and “L” (left) refers to genes that are transcribed in the negative sense. The diagram was built using the online platform available at http://bioinformatics.psb.ugent.be/webtools/Venn/. Click here for file

Additional file 4 Rio Negro virophage phylogenetic tree (A) (neighbor joining) and alignment (B) based on the predicted protein sequences of the capsid genes from RNV and other virophages. Click here for file
