# Supplementary figures and images for: Samba virus: a novel mimivirus from a giant rain forest, the Brazilian Amazon
Source: Virol J. 2014 May 14;11:95. doi: 10.1186/1743-422X-11-95 (PMC4113263; doi:10.1186/1743-422X-11-95)

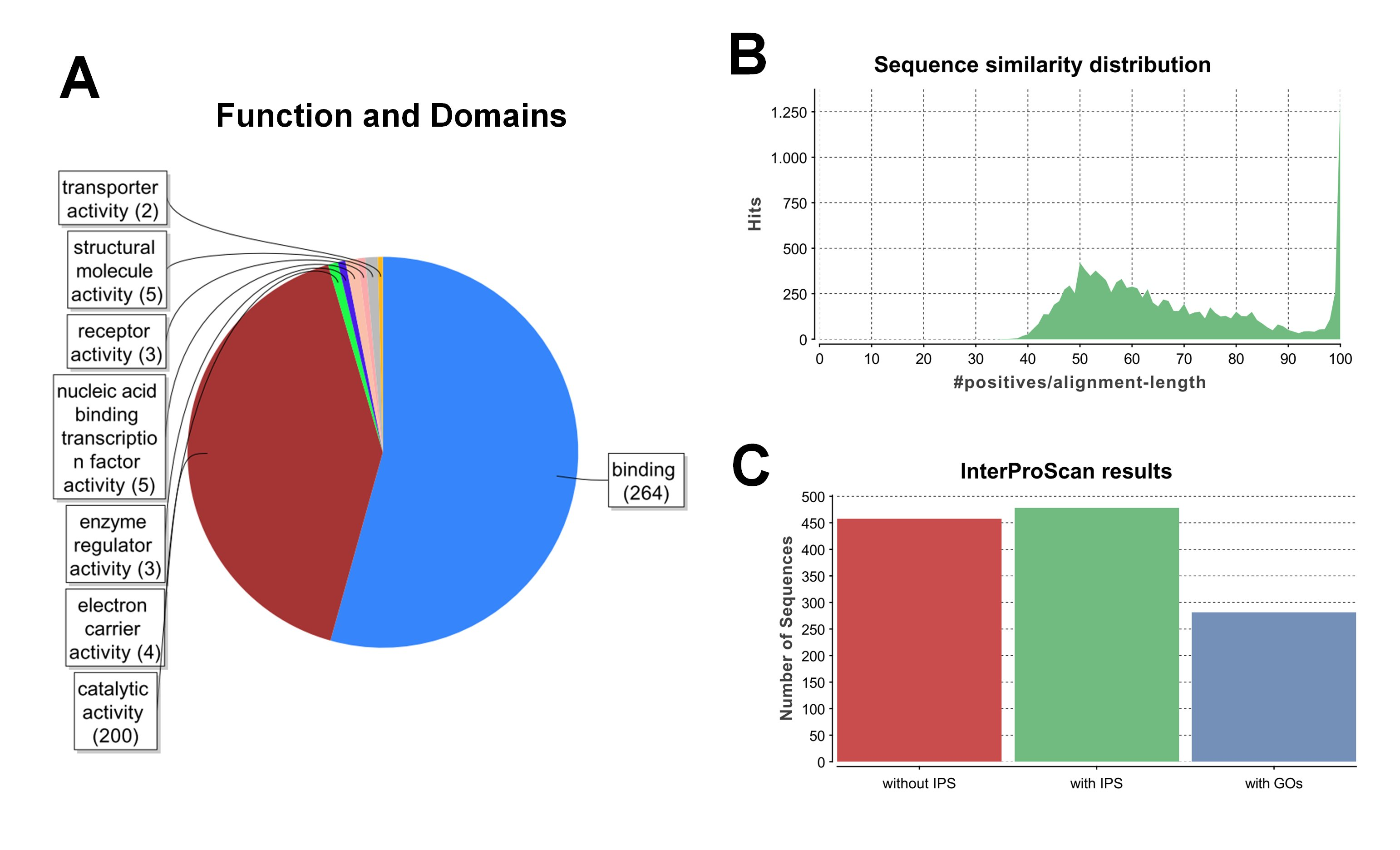

Supplement: Additional file 1 — Genomic data 2: Samba virus genome characterization performed using the java-based free software Blast2GO (available athttp://www.blast2go.com/b2ghome). (A) Graphical distribution of the functions and domains of predicted Samba virus genes. Most of the functions are related to catalytic and binding activities. (B) Graphical representation of the similarity of Samba virus genes to sequences available in the data bank of the Blast2GO program. The analysis showed a broad distribution of similarity ranging between 50-60%, with a peak near 100%. (C) Graphical depiction of Samba virus genes with or without functional annotation (IPS – InterProScan) and Samba genes grouped into orthologous groups (GO – Gene Ontology). [file 1743-422X-11-95-S1.tiff]

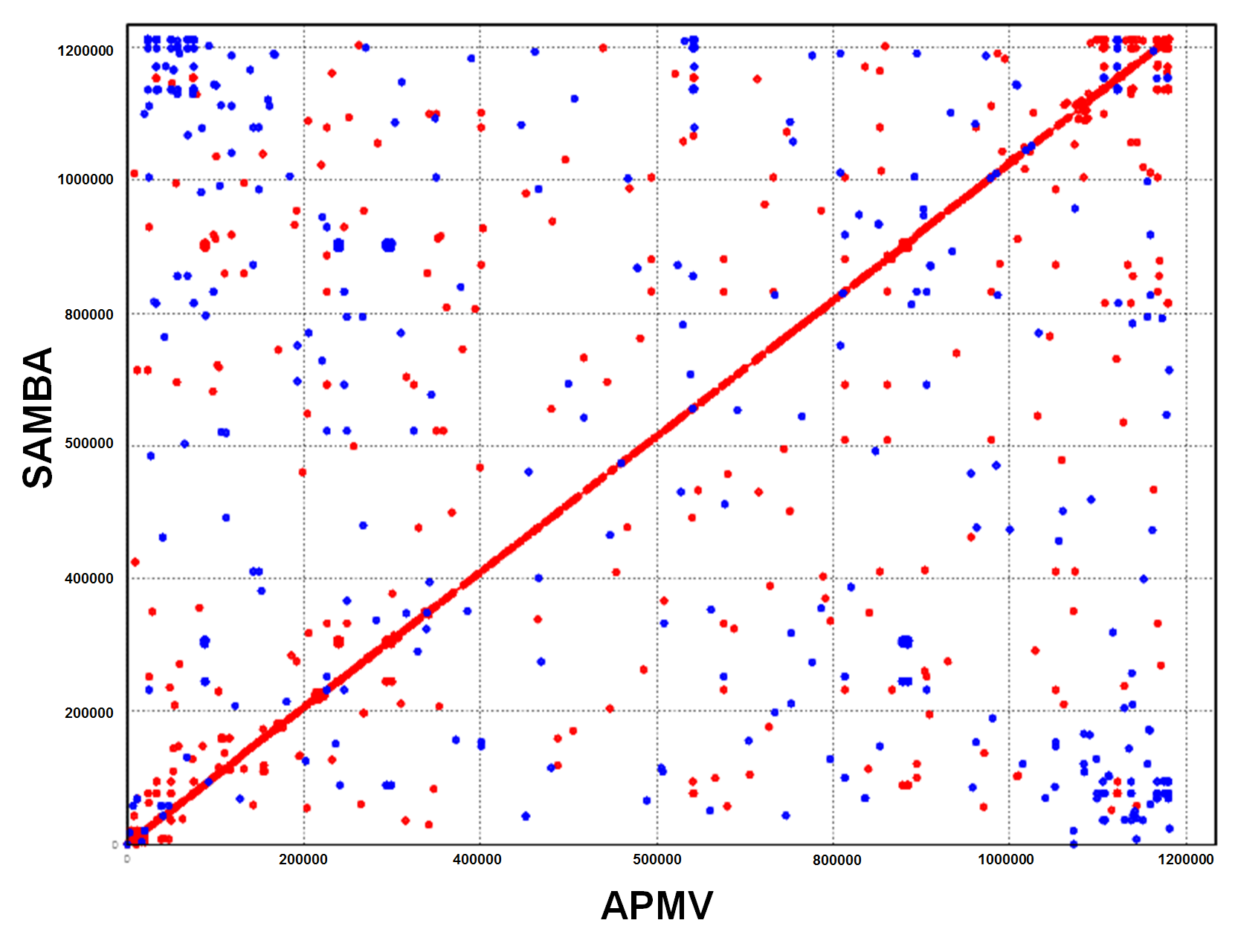

Supplement: Additional file 2 — Dotplot of SMBV vs. APMV ORFs – MUMMER 3.0 software. Dots represent the predicted ORFs. The red dots = plus-plus ORFs, and the blue dots = inverted ORFs. Although most of the SMBV genes are present in the same genome locus described for APMV, many ORFs are inverted or located in distinct loci, especially those present in terminal regions. [file 1743-422X-11-95-S2.tiff]

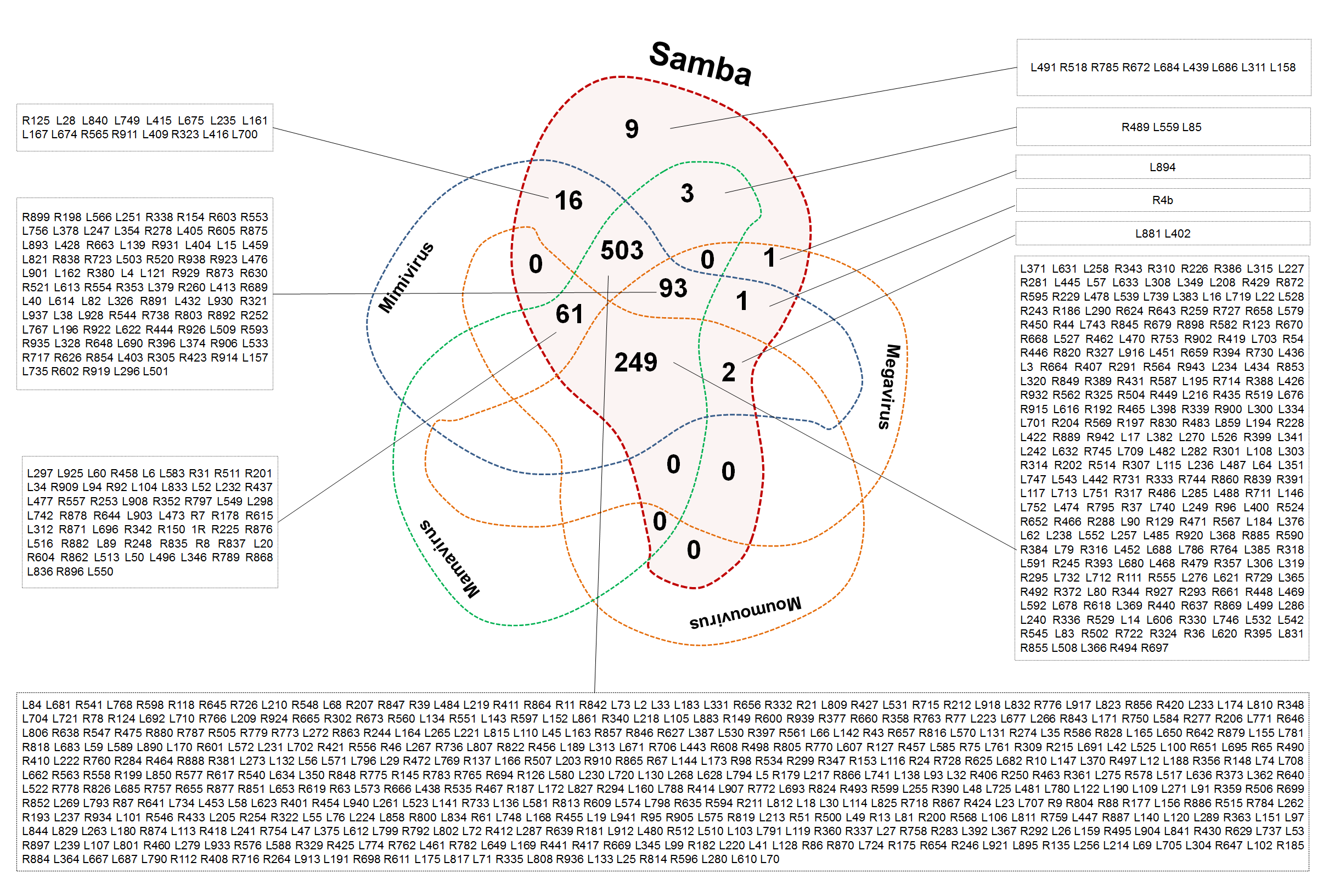

Supplement: Additional file 3 — Venn diagram: Venn diagram of predicted Samba virus genes relative to other Mimiviridae genomes. APMV – Acanthamoeba polyphaga mimivirus; Megavirus – Megavirus chilensis; Moumouvirus - Acanthamoeba polyphaga moumouvirus; Mamavirus – Acanthamoeba castellanii mamavirus. Boxes show each gene included in the intersections. “R” (right) refers to genes that are transcribed in the positive sense, and “L” (left) refers to genes that are transcribed in the negative sense. The diagram was built using the online platform available at http://bioinformatics.psb.ugent.be/webtools/Venn/. [file 1743-422X-11-95-S3.tiff]

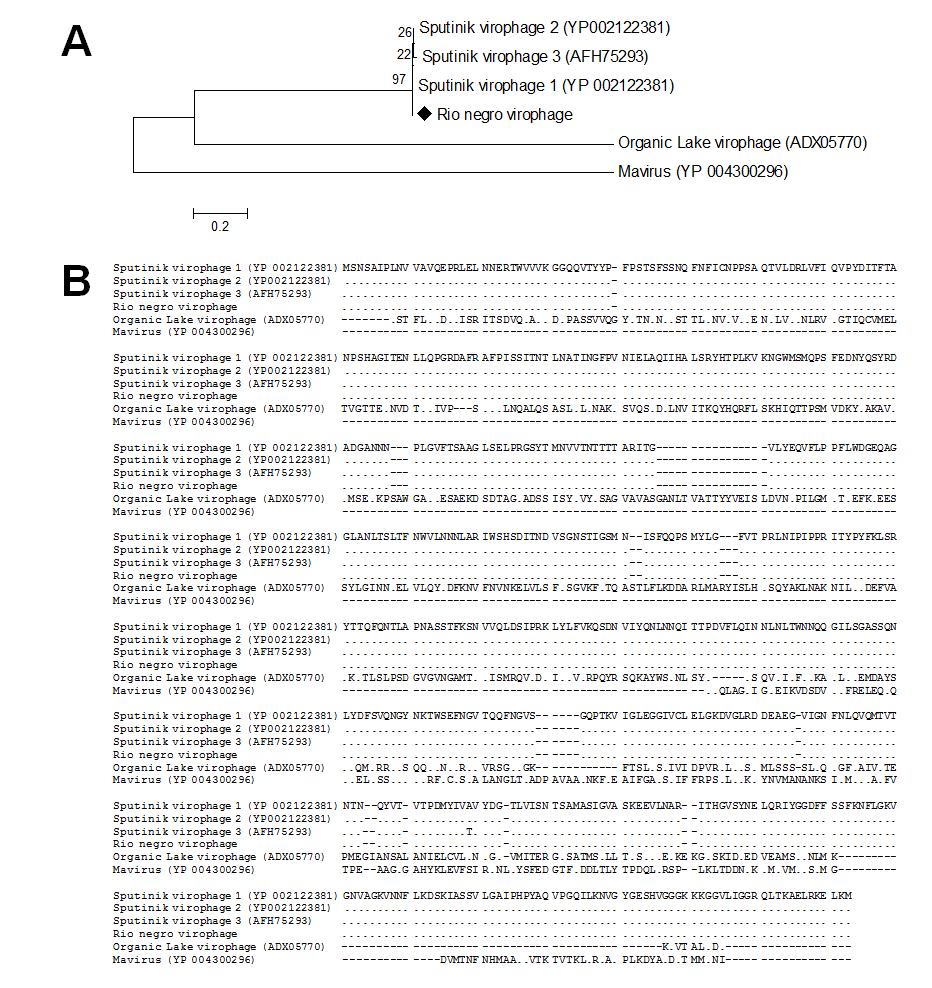

Supplement: Additional file 4 — Rio Negro virophage phylogenetic tree (A) (neighbor joining) and alignment (B) based on the predicted protein sequences of the capsid genes from RNV and other virophages. [file 1743-422X-11-95-S4.tiff]
